# Suppression of LPS-induced tau hyperphosphorylation by serum amyloid A

**DOI:** 10.1186/s12974-016-0493-y

**Published:** 2016-02-02

**Authors:** Jin Liu, Ding Wang, Shu-Qin Li, Yang Yu, Richard D. Ye

**Affiliations:** School of Pharmacy, Shanghai Jiao Tong University, Shanghai, 200240 China; Institute of Chinese Medical Sciences, University of Macau, Macau SAR, China

**Keywords:** Alzheimer’s disease, Serum amyloid A, Tau phosphorylation, Microglial activation

## Abstract

**Background:**

Accumulation of hyperphosphorylated tau is a major neuropathological feature of tauopathies including Alzheimer’s disease (AD). Serum amyloid A (SAA), an acute-phase protein with cytokine-like property, has been implicated in amyloid deposition. It remains unclear whether SAA affects tau hyperphosphorylation.

**Methods:**

Potential involvement of SAA in tau hyperphosphorylation was examined using intracerebral injection of SAA, and in *Saa3*^−/−^ mice receiving systemic administration of lipopolysaccharide (LPS). Induced SAA expression and microglial activation were evaluated in these mice using real-time PCR and/or immunofluorescence staining. Cultured primary neuronal cells were treated with condition media (CM) from SAA-stimulated primary microglial cells. The alteration in tau hyperphosphorylation was determined using Western blotting.

**Results:**

Saa3 is the predominant form of SAA proteins induced by LPS in the mouse brain that co-localizes with neurons. Overexpression of SAA by intracerebral injection attenuated tau hyperphosphorylation in the brain. Conversely, *Saa3* deficiency enhanced tau phosphorylation induced by systemic LPS administration. Intracerebral injection of SAA also induced the activation of microglia in the brains. IL-10 released to CM from SAA-stimulated microglia attenuated tau hyperphosphorylation in cultured primary neurons. IL-10 neutralizing antibody reversed the effect of SAA in the attenuation of tau phosphorylation.

**Conclusions:**

LPS-induced expression of SAA proteins in the brain leads to the activation of microglia and release of IL-10, which in turn suppresses tau hyperphosphorylation in a mouse model of systemic inflammation.

**Electronic supplementary material:**

The online version of this article (doi:10.1186/s12974-016-0493-y) contains supplementary material, which is available to authorized users.

## Background

Accumulating evidence suggests that neuroinflammation is a common pathological feature of tauopathy, characterized with intracellular accumulation of tau proteins in neurodegenerative disorders such as Alzheimer’s disease (AD) [1]. Microglia, the resident macrophages in the brain, are the primary immune cells responsible for the detection of invading pathogens and neuronal injuries in the central nervous system (CNS) [2]. Activated microglia are observed in the postmortem brain tissues of various human tauopathies including Alzheimer’s disease [3]. Microglial cells can be activated not only by injury and infection [4] but also by stimulators including amyloid beta (Aβ) [5] and serum amyloid A (SAA) [6]. Activated microglial cells in turn exhibit dramatic morphological changes, proliferate, migrate, and produce a variety of pro- and anti-inflammatory mediators [4, 7] such as interleukin-1 (IL-1), IL-6, and IL-18, which accelerate tauopathy including the formation of neurofibrillary tangles (NFTs) [8–11]. Although these findings suggest a possible link between neuroinflammation and tauopathy, there is little evidence for direct regulation of activated microglia in the pathological accumulation of the microtubule-associated protein tau.

Human SAA is a family of proteins consisting of SAA1, SAA2, and SAA4 [12]. Among these proteins, SAA1 and SAA2 are the major acute-phase proteins primarily synthesized by hepatocytes during acute-phase response [13]. However, extrahepatic production of SAA has been implicated as being more relevant to the pathogenesis of chronic inflammatory diseases including AD [13]. In mice, SAA is encoded by a family of three inducible genes, Saa1, Saa2, and Saa3, plus a constitutively expressed Saa4. The major site of their synthesis is the liver; however, Saa3 is also expressed in extrahepatic tissues in response to lipopolysaccharide (LPS) stimulation [14]. SAA has cytokine-like properties that modulate several cellular responses. SAA induces monocytes and neutrophils migration and stimulates the production of cytokines, chemokines, and matrix metalloproteinases (MMPs) [15–20]. Although the inducible SAA proteins are barely detectable in normal brains, SAA has been found in the brains of patients with AD [13, 21]. Moreover, SAA has been found to colocalize with Aβ deposits in AD brains [22], and the induction of a systemic acute-phase response in SAA transgenic mice enhances amyloid deposition [23]. Despite these findings, a correlation between SAA and tauopathy has not been established.

In this study, we found that the expression of the mouse SAA protein, Saa3, in the mouse brain was markedly increased in a LPS injection model of systemic inflammation. We evaluated the effects of SAA on tau phosphorylation in Saa3 gene knockout (*Saa3*^−/−^) mice with systemic LPS injection and in mice receiving intracerebral injection of SAA. The results have shown that the SAA proteins significantly attenuate tau hyperphosphorylation in these mouse models. Moreover, SAA has been found to regulate the activation of microglia in these mouse models. Finally, we have observed that IL-10 released from SAA-stimulated microglia attenuates tau hyperphosphorylation in neurons. Based on these findings, we postulate that SAA plays a role in tauopathy in part through the regulation of microglia activation.

## Methods

### Reagents

Primary antibodies used in this study are listed in Additional file 1: Table S1. The BCA protein assay kit and 4,6-diamidino-2-phenylindole (DAPI) were obtained from Beyotime Institute of Biotechnology (Nantong, Jiangsu, China). Mouse and rabbit control IgGs were purchased from Santa Cruz Biotechnology (Dallas, TX). IRDye®800CW secondary antibodies were from LI-COR (Lincoln, NE). Dulbecco’s modified Eagle’s medium (DMEM), neurobasal-A, and B-27® supplements were purchased from Life Technologies (Carlsbad, CA). Other chemicals were obtained from Sigma Chemical Company (St. Louis, MO).

### Animals

The *Saa3*^−/−^ (*Saa3* knockout) mice in C57BL/6 genetic background were obtained from the Knockout Mouse Project (KOMP) Repository (Davis, CA). Age- and sex-matched littermates were used in the experiments. The C57BL/6 mice were purchased from SLACCAS Laboratory Animal Co., Ltd (Shanghai, China). The generation of the Saa3 transgenic mice is described in Additional file 2. All mice were housed (four to five animals per cage) with a 12/12 h light/dark cycle, with ad libitum access to food and water. The housing, breeding, and animal experiments were in accordance with the National Institutes of Health Guide for the Care and Use of Laboratory Animals, with procedures approved by the Biological Research Ethics Committee of Shanghai Jiao Tong University.

### LPS administration

Lipopolysaccharide (LPS, from *Salmonella enterica* serotype Abortus equi, Sigma-Aldrich, Cat. No. L5886, Lot 032M4067) at a low concentration (5 mg/kg body weight) or a high concentration (15 mg/kg body weight) was intraperitoneally injected into 3-month-old C57BL/6 mice consisting of *Saa3*^−/−^ and their respective wild-type (WT) littermates (*n* = 4 per group; half males and half females). The mice received either LPS or an equal volume of normal saline. After 24 h, all mice were sacrificed by decapitation and their brains removed immediately. The hippocampi and cerebral cortices of the left hemisphere of the mouse brain were dissected, flash frozen in dry ice, and stored at −80 °C for biochemical analyses later. The right hemispheres of the mouse brains were fixed with 4 % paraformaldehyde in 0.1 M phosphate-buffered saline (PBS), followed by cryoprotection in 30 % sucrose. Coronal sections of 30-μm thickness were cut using a freezing sliding microtome. The sections were stored in glycol anti-freeze solution (ethylene glycol, glycerol, and 0.1 M PBS in 3:3:4 ratio) at −20 °C until immunohistochemical staining.

### Stereotaxic intracranial injection of SAA

Three-month-old C57BL/6 mice were randomly divided into control (saline) and SAA (recombinant human apo-SAA; Pepro Tech, Rocky Hill, NJ) (*n* = 4 in each group; half males and half females). Mice were anesthetized by intraperitoneal injection of 50 mg/kg pentobarbital sodium (Sigma) and then restrained onto a stereotaxic apparatus (RWD Life Science, Shenzhen, China). For stereotaxic intracranial injection of SAA, an earlier method was used [24]. After surgically exposed, the duramater and drilled holes in the skull, SAA (20 μg/8 μl) dissolved in saline was bilaterally injected into the left and right hippocampi (two sites, 4 μl per site), using a 10-μl Hamilton syringe with a 26-gauge needle. The injection rate was 0.5 μl/min, and the needle was kept in each site for an additional 3 min before a gentle withdrawal. The bregma coordinates were determined as follows: anterior, −2.0 mm; lateral, ±1.3 mm; vertical, −2.2 mm. As controls, the mice stereotaxically received equal volume of normal saline. Postoperatively, all mice were placed on heating pads (37 °C) until recovered from surgery. After 48 h, the injected mice were sacrificed by decapitation and the brain samples were prepared as with the LPS-treated mice described above.

For brain injection of SAA with IL-10 neutralizing antibody (BD Pharmingen, San Diego, CA), SAA (10 μg per site, two sites) with IL-10 neutralizing antibody (4 μg per site, two sites) were stereotaxically injected into the left and right hippocampi of the 3-month-old C57BL/6 mice (*n* = 4 in each group; half males and half females). As controls, the mice received an equal volume of SAA dissolved in saline (20 μg/8 μl) or normal saline alone (8 μl). After 48 h, the injected mice were sacrificed by decapitation and the brain samples were prepared as with the LPS-treated mice described above.

### Total RNA extraction and real-time PCR

Total RNA was extracted from the cortex and hippocampus of the mouse brain and primary cultures of neurons, astrocytes, and microglial cells using TRIzol reagent (Invitrogen). One microgram of the RNA was used for reverse transcription using the Reverse Transcription System A3500 kit (Promega, Madison, WI). The complementary DNA (cDNA) was subsequently subjected to Real-Time PCR to quantify the transcripts of TNF-α, IL-6, IL-10, Saa1, and Saa2, and Saa3 using SYBR® Green Real-time PCR Master Mix (TOYOBO, Osaka, Japan). The following primers were used: TNF-α (5′-TTC TCA TTC CTG CTT GTG G-3′; 5′-ACT TGG TGG TTT GCT ACG-3′), IL-6 (5′-CTT CTT GGG ACT GAT G-3′; 5′-CTG GCT TTG TCT TTC T-3′), IL-10 (5′-AGG GTT ACT TGG GTT GC-3′; 5′-TGA GGG TCT TCA GCT TC-3′), Saa1 (5′-TTG TTC ACG AGG CTT TC-3′; 5′-TTT GTC AGG CAG TCC AG-3′), Saa2 (5′-TGA TGC TGC CCA AAG G-3′; 5′-GCC AGG AGG TCT GTA GTA A-3′), and Saa3 (5′-CCT TCC ATT GCC ATC A-3′; 5′-GGG TCT TTG CCA CTC C-3′). The primers for the mouse housekeeping gene glyceraldehyde-3-phosphate dehydrogenase (GAPDH) were 5′-CCT TCC GTG TTC CTA CC-3′ and 5′-CAA CCT GGT CCT CAG TGT A-3′. PCR was performed according to the following conditions: 94 °C for 3 min, 40 cycles of denaturation at 94 °C for 30 s, annealing at 56 °C for 45 s, and extension at 72 °C for 30 s, followed by a final extension at 72 °C for 10 min. The quantitative fold changes in messenger RNA (mRNA) in each sample were normalized to GAPDH expression and calculated using the 2^exp(−ΔΔCt)^ method.

### Western blot analysis

Mouse brain tissues and cultured neuronal cells were homogenized in lysis buffer containing 50 mM Tris-HCl (pH 7.4), 8.5 % sucrose, 1.0 mM EGTA, 1.0 mM EDTA, 1.0 % sodium dodecylsulfate, 10 mM β-mercaptoethanol, protease, and phosphatase inhibitor cocktails (Sigma-Aldrich). Protein concentrations were determined using a BCA Protein Assay Kit (Beyotime Biotechnology, Nantong, China) and then heated in 5×SDS-PAGE loading buffer (Beyotime Biotechnology) at 99 °C for 7 min. Tissue and neuronal cell homogenates were separated on 10 or 12 % SDS-PAGE and transferred onto nitrocellulose membranes (Whatman Protran, Dassel, Germany). Blots were blocked with 5 % non-fat milk for 1 h at room temperature and incubated overnight at 4 °C with primary antibodies including Tau5 (1:2000); the anti-phospho-tau antibodies pS199 (1:2000), pS205 (1:2000), pS396 (1:2000), pS404 (1:2000), anti-Saa3(1:500), and anti-β-actin (1:10000), followed by the respective IRDye®800CW secondary antibodies (LI-COR Biosciences, Lincoln, NE). The membranes were scanned using the 800-nm channel of an Odyssey® CLX Infrared Imaging System. The immunoreactive bands were quantified using the NIH Image J software.

### Immunofluorescence staining

Sections of mouse brain were washed with 0.05 M Tris-buffered saline (TBS) (containing 0.05 M Tris buffer and 9 g/L NaCl, pH 7.4) for 15 min and then permeabilized with 0.1 % Triton X-100 in TBS for 15 min and blocked with 5 % normal goat serum (NGS) for 30 min at room temperature. The sections were incubated overnight at 4 °C with anti-Iba1 (1:500) in TBS with 5 % NGS. After washing with TBS for 15 min, the sections were incubated with Alex Fluor®568 donkey anti-rabbit antibody (1:500, Invitrogen) at room temperature for 1 h. Again, after three washes in TBS, the sections were stained for nuclei with 5 μg/ml of DAPI for 10 min at room temperature and mounted on glass slides. To identify the expression of Saa3 in neuron, microglia, or astrocyte, the brain sections were first stained with anti-MAP-2 (1:200), anti-CD11b (1:200), or anti-glial fibrillary acidic protein (GFAP)-Cy3^TM^ (1:500) antibody overnight and incubated in Alex Fluor®633 donkey anti-mouse or Alex Fluor®555donkey anti-rat IgG (1:500; Invitrogen) for 1 h. The sections were rinsed in TBS, stained with anti-Saa3 antibody (1:200) overnight at 4 °C, and incubated with an Alex Fluor®488 donkey anti-rabbit antibody (1:500; Invitrogen) for 1 h. Sections were counterstained with DAPI for 10 min, and then fluorescent confocal images were captured using a laser-scanning confocal fluorescence microscope (TCS SP8, Leica Microsystems, Wetzlar, Germany).

Images for the quantification of fluorescence intensity of Saa3, CD11b and GFAP were acquired at ×400 magnification. The relative immunofluorescence intensity of Saa3, CD11b, and GFAP was quantified using the ImageProPlus Software (Media Cybernetics, Silver Spring, MD). The results were expressed as mean ± SEM based on a minimum of three sections per animal and three 8-bit RGB digital images at the CA1 per animal (*n* = 4 mice per group).

To exclude obscure influence of Saa3 on microglial proliferation, the number of Iba1-positive cells was taken into consideration. Integrated optical density of red fluorescence (Iba1 positive area capture) was normalized against the integrated optical density of blue fluorescence (the DAPI positive area captured), using the Image ProPlus 6.0 software and expressed as mean ± SEM per group (three sections per animal, with images acquired at ×200 magnification; three 8-bit RGB digital images at the dentate gyrus (DG) and CA3 region per animal; *n* = 4).

### Primary neuronal cultures

Neuronal cultures were prepared in cortices from newborn (postnatal day 0) WT mouse pups as described before [25]. In brief, cerebral cortices were removed from the brains of mice, the meanings and microvessels were removed, and tissues were minced with a sterile razor blade. Tissues were digested with 0.025 % trypsin (Sigma) and 0.01 % DNase I (Sigma) at 37 °C for 10 min. The cell suspension was filtered through a 200-mesh sieve, and cells were plated on poly-d-lysine (Sigma)-coated 24-well plates at a density of 5 × 10^5^ cells per well. Two hours later, the DMEM medium was replaced with neurobasal medium containing 2 % B-27® supplements for 2 days. Culture medium were changed to neurobasal with 10 % fetal bovine serum (FBS) and 3 μg/ml cytosine-β-D-arabinofuranoside (Ara-C, Sigma) in the following 3 days and then again switched back to neurobasal medium containing 2 % B-27® supplements. Experiments were performed on days 7–8 after initiation of the culture.

### Primary microglial and astrocyte cultures

Microglial and astrocyte cultures were prepared from newborn (postnatal day 0) WT mouse pups as described before [6, 25, 26]. Specifically, separated cells were cultured in poly-D-lysine coated 75 cm^2^ flasks with DMEM medium (containing 10 % FBS, 100 U/ml penicillin, and 100 g/ml streptomycin sulfate). The medium was replenished on day 1 and day 3. On day 7, microglia cells in the culture flasks were shaken off at 260 rpm for 2.5 h, and the remaining astrocytes were maintained in DMEM with 10 % FBS. Experiments were performed after two passages of the cells.

### SAA and LPS treatment in vitro

Primary neuronal cells, astrocyte cells, and microglial cells were stimulated with LPS at indicated concentrations or with PBS for 24 h. Total RNA was extracted for real-time PCR to detect cell-specific expression of Saa3. For the analysis of the effect of SAA on neuronal tau phosphorylation, primary neuronal cells were treated with the recombinant human SAA (PeproTech, Rocky Hill, NJ; 0.5 μΜ for 3, 6 and 12 h), and the neuronal lysates were prepared for Western blot analysis as mentioned above.

### Preparation of conditioned media from microglia and astrocytes

The microglia cells and astrocyte were plated at a density of 1 × 10^6^ cells per well onto a 12-well plate for experiments involving the collection of conditioned media. The cells were treated with 0.5 μΜ SAA or PBS (control) for 6 h. The medium was removed and replaced with fresh DMEM. The cells were incubated for another 12 h, and the conditioned medium (CM) was collected. Half of the medium in the primary neurons was replaced with equal volume of CM from SAA-stimulated or PBS (control) cell culture. After 3, 6, and 12 h of incubation, neuronal lysate was prepared and blotted for Western analysis. To determine the effect of IL-10, an IL-10-neutralizing antibody (25 μg/ml; BD Pharmingen, San Diego, CA) was added to CM 30 min before application to primary cultures of neurons. After 12 h of incubation, neuronal lysate was prepared and Western blot analysis was conducted.

### ELISA

The IL-10 in the microglial conditioned medium was measured using enzyme-linked immunosorbent assay (ELISA) kit (eBioscience, San Diego, CA), according to the instructions of the vender. Briefly, the conditioned medium and mouse IL-10 standard samples were incubated in the IL-10 ELISA plates at 4 °C overnight. After washed plates three times with PBST (PBS + 0.5 % Tween-20) and blocked with 3 % BSA in PBS for 1 h at 37 °C, biotin-conjugate anti-mouse IL-10 antibody was incubated in the wells at 37 °C for 90 min. The plate was emptied and washed three times with PBST. Substrate solution was pipetted into each well after drying and incubated in 37 °C for 10 min. The enzymatic reaction was stopped by adding stop solution per well. The concentration of IL-10 was determined by measuring absorbance at 450 and 630 nm. All the experiments were done in three separate experiments.

### Statistical analysis

Data are presented as mean ± SEM from at least three experiments. One-way ANOVA followed by Newman-Keuls test or student’s *t* test was performed using the statistic software GraphPad Prism 5 (San Diego, CA). *p* values less than 0.05 was considered statistically significant.

## Results

### Systemic LPS administration enhances neuroinflammation and Saa3 expression in mouse brain

**Fig. 1:**
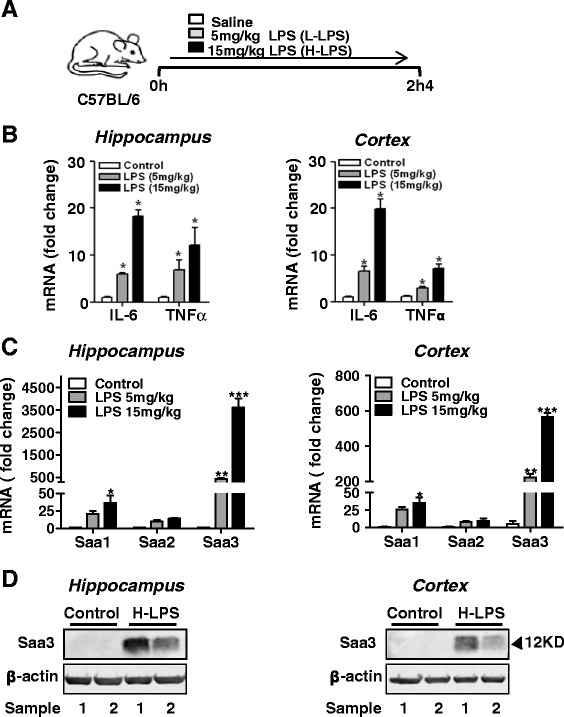
Induced expression of SAA and selected inflammatory cytokines in the mouse brain after systemic administration of LPS. **a** A schematic representation of experimental design. Three-month-old C57BL/6 mice were injected i.p. with LPS at either 5 mg/kg body weight (L-LPS) or 15 mg/kg body weight (H-LPS) or with same amount of saline (control). Twenty-four hours later, the mice were sacrificed and the hippocampus and cerebral cortex were collected for analysis. **b** Real-time PCR quantification of the transcripts for IL-6 and TNF-α in the tissue samples of the hippocampus (*left*) and cortex (*right*) 24 h after LPS or saline (control) administration. The relative transcript expression was calculated as 2-ΔΔ^CT^ and was normalized against saline controls. **c** relative mRNA levels of Saa1, Saa2, and Saa3 in extracts of hippocampus (*left*) and cortex (*right*) determined using the same procedure as in **b**. For each group, four mice were used and the data shown are the means ± SEM. *p < 0.05, **p < 0.01, ***p < 0.0001. **d** Two representative Western blots showing the 12-kDa Saa3 protein in the hippocampus (*left*) and cortex (*right*), following LPS (15 mg/kg body weight) stimulation for 24 h. An anti-Saa3 antibody was used for blotting

To evaluate the function of SAA in tau phosphorylation, SAA expression and distribution in the mouse brain was examined using a systemic inflammation model [27, 28]. Three-month-old C57BL/6 mice were given a single dose of LPS at 5 and 15 mg/kg through intraperitoneal injection. The control mice received an equal volume of normal saline. After 24 h, brain extracts from hippocampus and cortex were collected (Fig. 1a). Systemic administration of LPS induced neuroinflammation in the brain, as evidenced by a modest but significant increase in the transcripts of the pro-inflammatory cytokines IL-6 and TNF-α in the hippocampus and cortex (Fig. 1b). The effect of systemic LPS administration in SAA expression in the brain was next examined. All three inducible mouse *Saa* transcripts were elevated in the hippocampus and cortex (Fig. 1c). Of note, there was a 3500-fold increase in the hippocampus and a 600-fold increase in the cortex of the *Saa3* transcript in mice receiving 15 mg/kg of LPS compared to mice receiving normal saline (Fig. 1c). These results suggest that, in the mouse brain, Saa3 is the predominant form of SAA induced by LPS. The inducible expression of Saa3 was confirmed at the protein level using a specific antibody against Saa3, as shown in Western blots with two randomly chosen brain samples from mice receiving 15 mg/kg of LPS compared to those receiving normal saline (Fig. 1d).

**Fig. 2:**
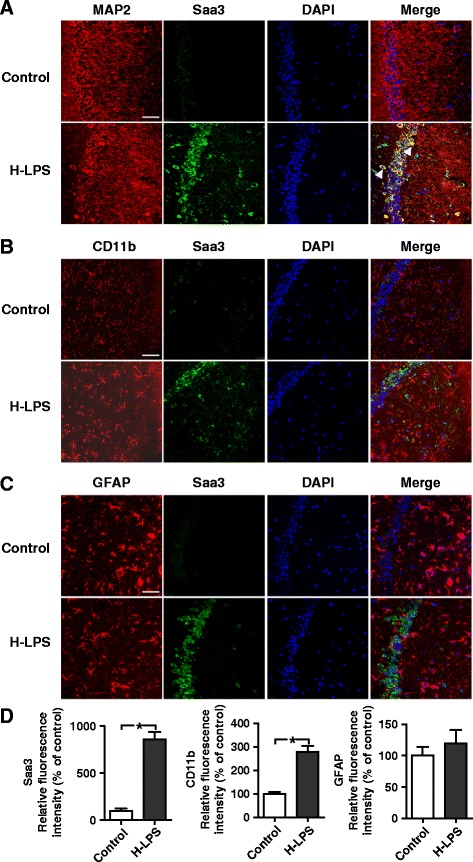
Immunofluorescence staining of Saa3 in the CA region of the hippocampus. Frozen sections from the left hemisphere of mice were stained for Saa3 protein using a rabbit anti-Saa3 antibody and Alexa Fluor 488-conjugated anti-rabbit IgG (*green fluorescence*). The sections were subsequently stained for neurons (anti-MAP2; **a**), microglia (anti-CD11b; **b**), or astrocytes (anti-GFAP; **c**), all in *red fluorescence* (see the “Methods” section for detail). Cell nuclei were stained with DAPI (*blue fluorescence*). Immunofluorescence was detected using a confocal laser-scanning microscopy. Images shown are representative of multiple experiments (*n* = 4 for each group; *scale bar*, 50 μm). *Arrowheads* in the merged image mark the positions of the double-stained cells. **d** quantification of the tissue expression level of Saa3, CD11b, and GFAP after LPS stimulation (15 mg/kg, 24 h). The results are expressed as the means ± SEM from at least three mice per group, each in duplicates or triplicates. *p < 0.05

In addition to immunoblotting, immunofluorescence staining was performed to confirm the increase in Saa3 expression and its distribution in the mouse brain. Staining of serial slices from WT mouse brain with an anti-Saa3 antibody and Alexa Fluor 488-conjugated secondary antibody identified a significant up-regulation of Saa3 (green fluorescence) in the CA1 (Fig. 2a–c) and DG (Additional file 3: Figure S1) regions of the hippocampus as well as in the cortex (Additional file 3: Figure S2) of mice receiving LPS, compared with mice receiving saline. To identify the cellular origin of Saa3, double immunostaining was performed for Saa3 and the cell-specific markers MAP2 (neurons), CD11b (microglia), and GFAP (astrocytes). The Saa3 protein was colocalized with MAP2 (Fig. 2a, Additional file 3: Figures S1A and S2A) and, to a lesser extent, with GFAP in the DG area of the hippocampus (Additional file 3: Figure S1C) and in the cortex (Additional file 3: Figure S2C). No Saa3 protein colocalization was visible in CD11b-positive cells, although induced expression of CD11b was evident in microglia from the LPS-injected mice (Fig. 2b, Additional file 3: Figures S1 and S2). These results show a much higher level of inducible Saa3 expression in neurons than in astrocytes, but not in microglia. Since Saa3 is reported to stimulate glial cells [6], its role in the activation of microglia and astrocytes after LPS injection was next examined. The expression of GFAP, a protein abundant in activated astrocytes, did not change in mice receiving LPS (Fig. 2c, d). In contrast, the CD11b-immunoreactive cells displayed a hypertrophic morphology, a sign of microglial activation. The expression of CD11b in these cells was significantly higher than that in the control (saline) samples (Fig. 2c, d). Altogether, these results demonstrate that Saa3 is the major form of SAA proteins induced by LPS in mouse neurons and astrocytes.

### SAA deficiency potentiates tau phosphorylation in LPS-injected mice

**Fig. 3:**
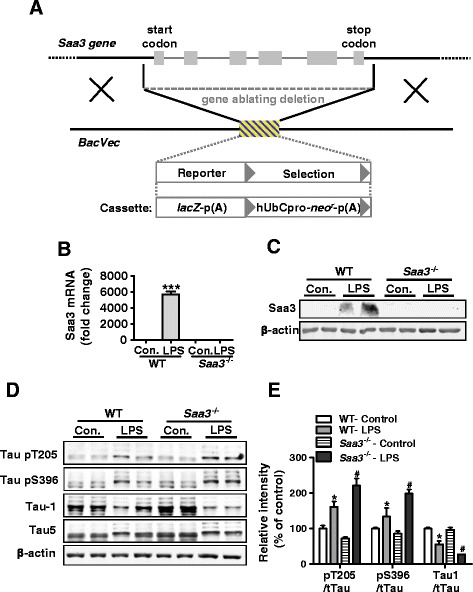
Genetic deletion of *Saa3* promotes LPS-induced tau hyperphosphorylation. **a** The Saa3 gene knockout construct based on information provided by www.komp.org. *Gray boxes* are exons, and the hatched area denotes expression-selection cassette. **b**, **c** Absence of LPS-induced expression of the *Saa3* transcript (**b**) and protein (**c**), in the *Saa3* ^−/−^ mice. The brain tissue from the hippocampi of WT and *Saa3* ^−/−^ mice was collected 24 h after receiving LPS (15 mg/kg) or saline (control). Tissue homogenate was made for real-time PCR (**b**) and Western blotting (**c**). Representative blots were probed with an anti-Saa3 antibody. β-actin was used as a loading control.***p < 0.01 compared with mice receiving normal saline (*n* = 4). **d** Immunoblots of protein extracts from the hippocampi of WT and *Saa3* ^−/−^ mice receiving LPS (15 mg/kg, 24 h) or saline (control). Representative blots were probed with anti-phospho-tau antibodies recognizing phosphorylated tau at Thr205 and Ser396 and with antibodies against non-phosphorylated tau (Tau1) and total tau (Tau5), respectively. β-actin was used as a loading control. **e** Quantification of the immunoblots. The relative phosphorylation level of tau in each sample was normalized against the integrated density of total tau (Tau5) present in each sample. All data shown are mean ± SEM, with four mice in each group; *p < 0.05 compared with WT controls; ^#^ *p* < 0.05 compared with WT receiving LPS stimulation

To determine whether the elevated Saa3 expression plays a role in neuroinflammation and AD development, *Saa3* gene knockout (*Saa3*^−/−^) mice were generated (Fig. 3a). The success of *Saa3* deletion was functionally confirmed in mice receiving systemic LPS administration, which led to elevated Saa3 expression in WT but not the *Saa3*^*−/−*^ mice (Fig. 3b, c). These mice were next compared for the extent of tau hyperphosphorylation, a major pathological feature of AD [1]. The contents of total tau (detected by the Tau5 antibody), non-phosphorylated tau (detected by the Tau1 antibody), and phosphorylated tau at two AD-related amino acid positions (detected by antibodies against pT205 and pS396, respectively) were determined in the hippocampus of *Saa3*^*−/−*^mice and their WT littermates that received LPS or saline. As shown in Fig. 3d, e, there was an increase in tau phosphorylation at T205 and S396 in WT and *Saa3*^*−/−*^ mice receiving LPS injection, compared to mice receiving saline injection. A decrease in the level of non-phosphorylated tau in LPS-injected mice was also evident when normalized against total tau (Tau5). When the *Saa3*^−/−^ mice were compared with their WT littermates, the *Saa3*^−/−^ mice showed an additional increase in tau phosphorylation at T205 and S396, along with a more evident decrease in non-phosphorylated tau between the saline-injected mice and LPS-injected mice (Fig. 3d, e). The results were further confirmed by immunofluorescence staining analysis. As expected, after systemic LPS treatment, the expression of phosphorylated tau at T205 had an additional increase in neuronal cells from the *Saa3*^−/−^ mouse (Additional file 3: Figure S3), but not in astrocytes (Additional file 3: Figure S4). These findings suggest a role for Saa3 in restraining tau hyperphosphorylation induced by systemic LPS administration.

### Transgenic expression of Saa3 attenuates tau hyperphosphorylation

To further evaluate the potential involvement of Saa3 in regulating tau hyperphosphorylation, transgenic mice expressing Saa3 were generated. Based on our finding that induced Saa3 expression was seen mainly in neurons, the *Saa3* transgenic (*Saa3*-Tg) mice were prepared using the neuron-specific synapsinI (SYNI) promoter (Additional file 3: Figure S5A–B; see Additional file 2 for detail). Transgenic expression of Saa3 increased the basal and inducible *Saa3* mRNA level (Additional file 3: Figure S5C). Of note, the basal and inducible expression of Saa3 was also enhanced at the protein level in the *Saa3*-Tg mice (Additional file 3: Figure S5D–E). To determine whether Saa3 was secreted by neurons, primary neuronal cultures prepared from newborn *Saa3*-Tg mouse pups and their WT littermates were stimulated with 0.1 or 1 μg/ml of LPS for 24 h. The ELISA result showed a higher level of Saa3 protein in the culture medium from *Saa3*-Tg mice than non-Tg mice, either with or without LPS stimulation (Additional file 3: Figure S5F). These results validate the transgenic expression of Saa3 in the mouse brain.

To investigate whether transgenic expression of Saa3 affects tau phosphorylation, the level of total tau and phosphorylated tau at T205 and S396 was determined in *Saa3*-Tg mice and non-Tg controls following systemic LPS administration (Additional file 3: Figure S6). When normalized against total tau, an increase in tau phosphorylation at T205 and S396 was observed in non-Tg mice injected with LPS compared with that of saline controls. In the *Saa3*-Tg mice, however, the level of LPS-induced tau phosphorylation at T205 and S396 was lower than that of the non-Tg mice.

### Intracerebral injection of SAA attenuates tau hyperphosphorylation

**Fig. 4:**
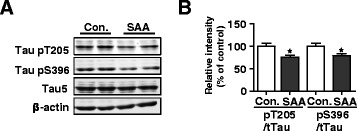
Intracerebral SAA injection attenuates tau hyperphosphorylation in mouse brain. **a** The tau phosphorylation level from hippocampal extracts was determined by immunoblot analysis with anti-phospho-tau antibodies at Thr205 and Ser396 following stereotaxical injection of SAA or saline (control) into the mouse brain. Total tau level and β-actin was also examined. **b** The relative tau phosphorylation level for each sample was normalized the integrated density of total (Tau5) between samples. All data shown are mean ± SEM; *n* = 4 mice in each group; *p < 0.05 versus control group

Intracerebral injection has been used widely to assess the effects of drugs and cell-derived factors in brain functions. To further evaluate the potential involvement of Saa3 in regulating tau hyperphosphorylation and to determine whether this effect is unique to Saa3 or generally applicable to other acute-phase SAA proteins, mice were given hippocampal injection of recombinant SAA or saline. The level of total tau and phosphorylated tau at T205 and S396 was determined in the hippocampus of mice receiving SAA or saline injection. As shown in Fig. 4, when normalized against total tau, a decrease in tau phosphorylation at T205 and S396 was observed in mice receiving SAA injection compared with mice receiving saline.

### SAA induces microglia activation in mice receiving intracerebral injection of SAA

**Fig. 5:**
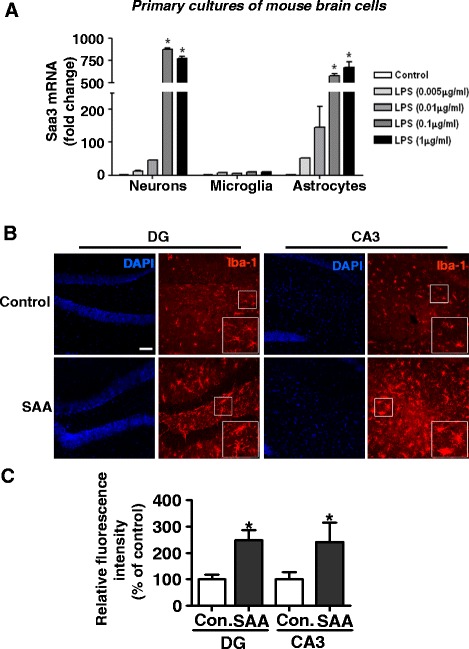
Microglia activation after intracerebral injection of SAA. **a** LPS-induced Saa3 expression in primary cultures of neurons and astrocytes but not microglia. Quantitative real-time PCR detection of Saa3 transcript using primary cultures of neurons, microglia, and astrocytes after LPS stimulation (5, 10, 100, and 1000 ng/ml) for 24 h. Shown are relative mRNA expression levels based on three independent experiments; mean ± SEM; *p < 0.05 versus controls). **b** Iba1 stain of the dentate gyrus and CA3 regions of the mouse brain, showing increased microglia activation, based on Iba 1 expression, in mice receiving brain injection of 20 μg SAA compared to those receiving normal saline. **c** Quantification of Iba1 density, based on images in **b** and similar samples, was expressed as means ± SEM (three sections per animal; three images at dentate gyrus and CA3 per animal; *n* = 4 per group; *p < 0.05 compared with the controls (normal saline). *Scale bar*, 75 μm

It was shown in Fig. 2 that Saa3 was found primarily in neurons, whereas its expression was absent from microglia. This finding was confirmed in primary cultures of brain cells. At the mRNA level, marked induction of the *Saa3* transcript was observed in mouse neurons and astrocytes, but not in microglia (Fig. 5a). Experiments were conducted to determine whether microglia could respond to SAA and play a role in neuroinflammation, as suggested previously [6]. As shown in Fig. 5b and quantified in Fig. 5c, microglia was markedly activated in the hippocampus of mice receiving intracerebral injection of SAA, compared with mice receiving saline only. This result is consistent with our previous report that SAA could induce the activation of microglia in primary culture [6], suggesting a potential role for SAA in microglial activation.

### IL-10 released by SAA-stimulated microglia reduces tau phosphorylation in primary cultures of neurons

**Fig. 6:**
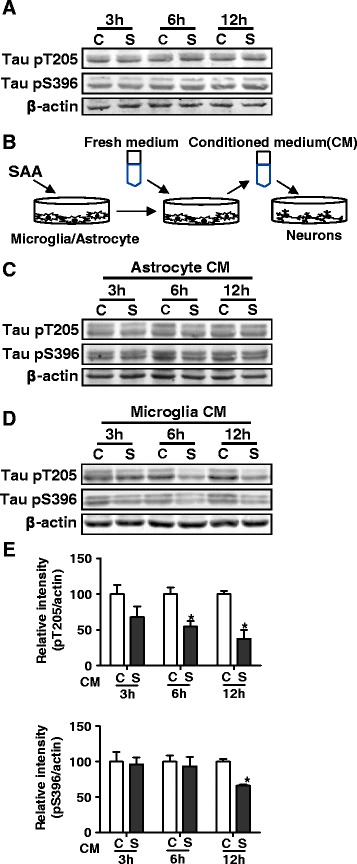
Soluble factors in SAA-stimulated microglia culture medium are responsible for the reduced tau phosphorylation in neurons. **a** Absence of a direct effect of SAA in tau phosphorylation in neurons. Primary cultures of mouse neurons were stimulated with 0.5 μM of SAA for 3, 6, and 12 h. The cells were collected, and tau phosphorylation was determined with Western blotting using specific antibodies recognizing phosphorylated tau at T205 and S396. β-actin was a loading control. **b** Experimental scheme showing stimulation of primary astrocytes and microglia with SAA (0.5 μM) or PBS for 6 h, removal of medium, addition of fresh medium and culture for 12 h, and collection of conditioned medium (CM) for stimulation of primary cultures of neurons. **c** Western blots showing the level of tau phosphorylation at T205 and S396 after stimulation with 50 % of CM from cultured astrocytes, as in **b. d** Western blots showing the level of tau phosphorylation at T205 and S396 after stimulation with CM from cultured microglia. The levels of tau phosphorylation at T205 and S396, as in **d** and similar samples receiving CM from cultured microglia, were quantified using densitometry, normalized against β-actin, and shown in bar graphs in (**e**; mean ± SEM shown, *p < 0.05; *n* = 3 independent cultures)

Experiments were conducted to determine whether SAA acts directly on neurons to affect tau phosphorylation. As shown in Fig. 6a, SAA treatment of primary neurons in culture did not have a significant effect on tau phosphorylation, suggesting that SAA might influence tau phosphorylation through an indirect mechanism. Since SAA is known to regulate microglial activation, it was postulated that SAA-treated microglia might release mediator(s) that affect tau hyperphosphorylation. To test this possibility, primary neurons from the mice were cultured with conditioned media (CM) of microglia or astrocyte collected after exposure to SAA (Fig. 6b). As expected, these cells showed reduced tau phosphorylation in the presence of the CM from SAA-treated microglia (Fig. 6c). In comparison, the level of tau phosphorylation was not changed when neurons were cultured with CM from SAA-treated astrocyte (Fig. 6d). This finding confirms our hypothesis that SAA-stimulated microglia release mediator(s) that affects tau phosphorylation.

**Fig. 7:**
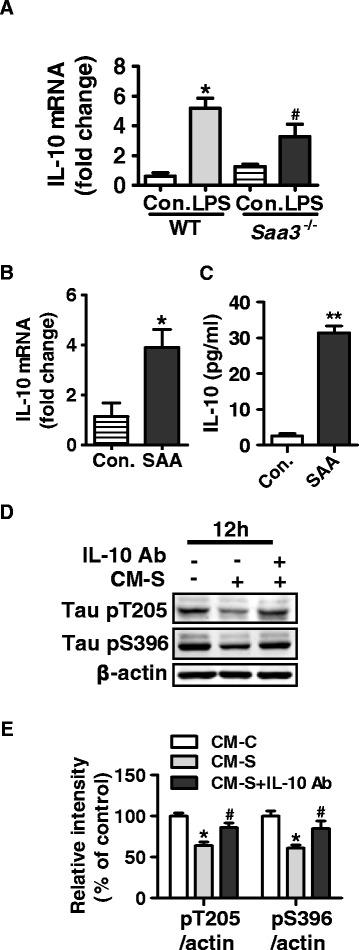
IL-10 is required for SAA-induced reduction of tau phosphorylation. **a** Quantification of real-time PCR for IL-10 transcripts from the hippocampi of WT and *Saa3* ^−/−^ mice receiving LPS (15 mg/kg, 24 h) or saline (control). Plotted is the relative mRNA expression calculated as 2-ΔΔ^CT^ method and normalized to controls. **b** The transcript of IL-10 detected by real-time PCR in brain extracts of hippocampus 48 h after hippocampal injection of mice with SAA (20 μg in 8 μl) or equal volume of normal saline. **c** The level of IL-10 protein in the CM of primary microglia receiving SAA (0.5 μM) or PBS, using the stimulation scheme described in Fig. 6b. **d** Effect of neutralizing antibody for IL-10 on tau phosphorylation at T205 and S396 after incubation with CM from SAA-stimulated primary microglia (CM-S). Primary cultures of neurons were stimulated with 50 % CM-S (+) or control (without SAA stimulation, CM-C) for 12 h. The IL-10 neutralizing antibody (25 μg/ml) was added to CM-S 30 min before application to primary cultures of neurons. The level of tau phosphorylation at T205 and S396 was determined by Western blotting using specific anti-phospho-tau antibodies. β-actin was a loading control. **e** Quantification of the levels of tau phosphorylation was conducted using densitometry. The results are shown in *bar graph* as mean ± SEM, based on three independent experiments. *p < 0.05 compared with CM-C stimulated samples; ^#^ *p* < 0.05 compared with CM-S stimulated samples without neutralizing antibody

We found that the level of IL-10 mRNA was lower in *Saa3*^*−/−*^mice than in their WT littermate controls following LPS stimulation (Fig. 7a). Consistent with this finding, the mRNA level of IL-10 was increased after intracerebral injection of SAA (Fig. 7b). There was also a remarkable increase in IL-10 protein production in microglial CM after SAA stimulation (Fig. 7c). To confirm that IL-10 was the mediator of interest, an anti–IL-10 monoclonal antibody was included in the culture. Notably, the effect of microglial CM on tau phosphorylation was partially reversed with the anti–IL-10 antibody (Fig. 7d), suggesting involvement of IL-10 in the regulation of tau phosphorylation.

**Fig. 8:**
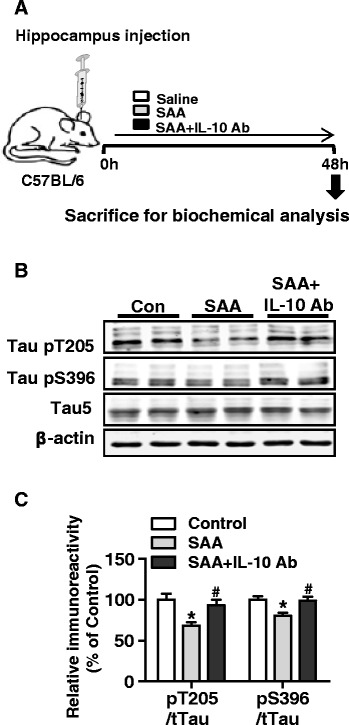
SAA-induced reduction of tau phosphorylation is blocked by neutralizing IL-10. **a** A schematic representation of experimental design. SAA (20 μg) was stereotaxically injected into the left and right hippocampus with the IL-10 neutralizing antibody (8 μg) or saline (4 μl per site). Assays were conducted after 48 h. **b** Representative blots were probed with anti-phospho-tau antibodies recognizing phosphorylated tau at Thr205 and Ser396 and total tau (Tau5), respectively. β-actin was used as a loading control. **c** Quantification of the relative phosphorylation level of tau in each sample, normalized against the integrated density of total tau (Tau5) in each sample. All data shown are mean ± SEM, with four mice in each group; *p < 0.05 compared with mice receiving saline controls; ^#^ *p* < 0.05 compared with mice receiving SAA

To further explore the potential involvement of IL-10 in the SAA-induced reduction of tau hyperphosphorylation, mice were given hippocampal injection of recombinant SAA with or without the IL-10 neutralizing antibody. The control mice received hippocampal injection of saline. The level of total tau and phosphorylated tau at T205 and S396 was determined. As shown in Fig. 8, when normalized against total tau, a decrease in tau phosphorylation at T205 and S396 was observed in mice receiving intracerebral SAA injection compared with mice receiving saline. The inclusion of the IL-10 neutralizaing antibody with SAA injection reversed the decrease in tau phosphorylation at T205 and S396.

## Discussion

The present study brings two new findings. One is a suppressive function of brain SAA in tau hyperphosphorylation, and the other is the involvement of microglia in this function. The majority of the published studies show that intracellular neurofibrillary tangles (NFTs) often accompany microglia activation [29], suggesting a possible link between activated microglia and tau phosphorylation. However, except studies showing that altered microglia activation play a role in modulating tau hyperphosphorylation within neurons via the CX3CL1-CX3CR1 interaction [30, 31], there has been no direct evidence for an effect of microglia activation in tau hyperphosphorylation. Our previous study found that SAA induces changes in the morphology, survival, and cytokine production in murine microglia, indicating that it plays a role in the activation of microglia [6]. The present study demonstrated for the first time that SAA is involved in the regulation of microglia activation and in modulating tau phosphorylation using different mouse models. Our experimental data confirmed that IL-10 released from the SAA-stimulated microglia could affect tau hyperphosphorylation in cultured primary neurons. Collectively, these results provide new evidence for an indirect mechanism by which SAA modulates AD-related pathologies.

In the present study, we evaluated the effect of SAA on tau hyperphosphorylation using the mouse model of systemic LPS administration. LPS has been widely utilized in mouse models to induce neuroinflammation via either systemic administration or direct injection into the brain [30, 32–35]. Notably, several recent studies showed that the acute effect of LPS can induce tau hyperphosphorylation in the mouse brain and worsen the tau pathology in Tau-Tg mice or 3xTg mice [30, 32, 36–39]. Consistent with these published studies, we found that a single dose of LPS given peripherally is sufficient to induce neuroinflammation and tau hyperphosphorylation. Furthermore, using *Saa3*^−/−^ mice, our studies demonstrate that the LPS-induced changes in tau phosphorylation are altered in *Saa3*-deficient mice. These findings provide first evidence for a potential role of the SAA proteins in LPS-induced tau hyperphosphorylation.

The present study also provides the first documentation of local expression and distribution of SAA isoforms in the mouse brain. We found that Saa3 is the predominant SAA isoform induced by systemic LPS administration in the mouse brain. As a result of this finding, *Saa3* was chosen for further analysis using a gene knockout approach. Because Saa3 is less well characterized for its regulatory functions compared to the acute-phase SAA of human origin (SAA1 and SAA2), we have included in our study a human SAA protein in intracerebral injection and in the stimulation of cultured brain cells. Both experiments have given results that complement each other and together support a regulatory role of SAA in tau phosphorylation. We have also found that a significant amount of the induced Saa3 protein is in neurons and, to a much lesser extent, astrocytes in the DG region of hippocampus and in the cortex. However, SAA expression is negligible in microglia under the same experimental conditions. This finding is unexpected as previously published studies have shown that macrophages, which share many properties with microglia, are a source of SAA proteins [40]. The discrepancy may result from the efficiency of Saa3 translation in different types of cells and from the difference in the in vivo (brain sections) and in vitro (cultured cells) experimental conditions.

Despite the lack of SAA production by microglia, these cells can nevertheless respond to SAA stimulation with the release of factors that regulate tau hyperphosphorylation in neurons. In comparison, neurons fail to respond to SAA directly in assays for tau phosphorylation, suggesting that the neuron-derived SAA acts through a paracrine mechanism that involves a mediator. IL-10 is an anti-inflammatory cytokine known for its inhibition of Aβ- and LPS-induced pro-inflammatory cytokines and chemokines in the brain [41]. Two studies have shown that down-regulation of tau phosphorylation is accompanied by up-regulation of IL-10 [42, 43], suggesting a possible link between IL-10 and tau phosphorylation. In the present study, we demonstrated that IL-10 released by SAA-stimulated microglia attenuates tau phosphorylation in neurons, and neutralizing IL-10 could reverse the decrease of tau phosphorylation induced by intracerebral SAA injection in mice. These results provide additional evidence for an effect of IL-10 on tau phosphorylation. Therefore, IL-10 serves to mediate the function of SAA, which acts indirectly. A previous example of the indirect action is reported by Harrison et al., who document that CX3CL1 is produced by neurons in the brain and signals through CX3CR1, which is expressed in microglia [44, 45]. More interestingly, Kevin Nash et al. found that overexpression CX3CL1 in the rTg4510 mouse model directly suppresses tauopathies [46]. Together, these results provide evidence for the indirect regulatory mechanism of microglial cells in modulating tau phosphorylation and indicate that glial cell regulation may be a potential therapeutic strategy for tauopathies.

Previous study from our laboratory demonstrated that SAA administration could induce significant activation of primary microglial cells in culture [6]. Consistent with this finding, in the present study, stereotaxic hippocampal injection of SAA induced microglia activation in the mouse brain. These findings suggest that the regulatory function of SAA may depend on the activation states of microglia. Both the present study and our previous study [6] show that SAA can induce the release of pro-inflammatory cytokines including IL-1β, IL-6, and TNF-α, as well as the anti-inflammatory cytokine IL-10. The pro-inflammatory cytokines and anti-inflammatory cytokines released from SAA-stimulated microglia may contribute to the homeostatic function of SAA in the brain. It is through the action of the anti-inflammatory cytokine IL-10 that SAA exerts its regulatory effect on tau phosphorylation. Future studies will be required to illuminate whether and how other cytokines released by activated microglia also contribute to the regulation of tau phosphorylation and how SAA-mediated activation of microglia plays a role in this process.

## Conclusions

The present study demonstrates that SAA plays a role in regulating tau hyperphosphorylation in mice. In these models, SAA attenuates tau hyperphosphorylation induced by systemic LPS administration, as evidenced by elevated tau phosphorylation in Saa3 deficient mice. This effect of SAA is not exerted directly on neurons; instead, IL-10 released by the SAA-activated microglia serves to inhibit tau hyperphosphorylation. Our results suggest that SAA is a potential target for therapeutic intervention of AD.

## Additional files

Additional file 1: Table S1. The primary antibodies used in this study.This table lists the names, specificities and sources of the primary antibodies used in the study. Where applicable, phosphorylation sites recognized by the antibodies are given.

Additional file 2: **Supplementary materials.** This file contains detailed materials and methods used in the present study but not shown in the main text of the paper.

Additional file 3: **This contains Figures S1–S6.** This file contains 6 supplementary figures, listed as Figures S1 to S6, that provide the reader with additional information related to the main text of the paper.

## Abbreviations

AD: Alzheimer’s disease
Aβ: amyloid beta
CM: conditioned medium
CNS: central nervous system
DAPI: 4,6-diamidino-2-phenylindole
DMEM: Dulbecco’s modified Eagle’s medium
GFAP: glial fibrillary acidic protein
IL-1: interleukin-1
LPS: lipopolysaccharide
NFTs: neurofibrillary tangles
NGS: normal goat serum
SAA: serum amyloid A
SYNI: synapsin I
TBS: Tris-buffered saline
WT: wild type
