# Supplementary material for: Suppression of LPS-induced tau hyperphosphorylation by serum amyloid A
Source: J Neuroinflammation. 2016 Feb 2;13:28. doi: 10.1186/s12974-016-0493-y (PMC4736117; doi:10.1186/s12974-016-0493-y)
Supplement: Supplementary file 1 — The primary antibodies used in this study.This table lists the names, specificities and sources of the primary antibodies used in the study. Where applicable, phosphorylation sites recognized by the antibodies are given. [file 12974_2016_493_MOESM1_ESM.docx]

**Additional file 1: Table S1. The primary antibodies used in this study**

| **Antibody** | **Type** | **Specificity** | **Phosphorylation sites** | **Reference/Source** |
| --- | --- | --- | --- | --- |
| Tau5 | Mono- | Tau (total) |  | Invitrogen, Carlsbad, CA, USA |
| Tau1 | Mono- | Tau (non-phos.) | Ser198/Ser199/Ser202 | Millipore, Temecula, CA, USA |
| pT205 | Poly- | P-tau | Thr205 | Invitrogen |
| pS396 | Poly- | P-tau | Ser396 | Invitrogen |
| SAA3 | Poly- | SAA3 |  | ABclonal Technology, Wuhan, China |
| MAP-2 | Mono- | MAP-2 |  | Bio-world, Louis Park, MN, USA |
| CD11b | Mono- | CD11b |  | BD Biosciences Pharmingen, San Diego, CA |
| Iba1 | Poly- | Iba1 |  | Wako, Osaka, Japan |
| GFAP−Cy3^™^ | Mono- | GFAP |  | Sigma-Aldrich, St Louis, MO, USA |
| Anti-β-actin | Mono- | β-actin |  | Sigma |
| Anti-GAPDH | Poly- | GAPDH |  | GoodHere Technology, Hangzhou, China |
