# Supplementary material for: Suppression of LPS-induced tau hyperphosphorylation by serum amyloid A
Source: J Neuroinflammation. 2016 Feb 2;13:28. doi: 10.1186/s12974-016-0493-y (PMC4736117; doi:10.1186/s12974-016-0493-y)
Supplement: Supplementary file 2 — Supplementary materials. This file contains detailed materials and methods used in the present study but not shown in the main text of the paper. [file 12974_2016_493_MOESM2_ESM.docx]

### Supplementary Materials

**Supplementary Methods**

**Animals**

**Preparation of the *Saa3* transgenic mice.** Mice expressing the *Saa3* transgene (*Saa3*-Tg) were generated at Cyagen Biosciences, Inc. (Guangzhou, China). In brief, the cDNA of murine Saa3 under the control of the neuron-specific rat-synapsinI (SYNI) promoter was cloned into the pRP.Des2d vector (Fig.S5A). Transgenic mice in C57BL/6 background were produced and identified by PCR with the forward and reverse primers 5’-CTG CCTA AAA GAT ACT GAA CCC and 5’-ACG GAT CCT TAT CGA TTT TAC CA, respectively. The internal control was identified by PCR with the forward and reverse primers 5’-ACT CCA AGG CCA CTT ATC ACC and 5’-ATT GTT ACC AAC TGG GAC GACA. The transgenic PCR product size was 312bp, and the internal control PCR product size was 413bp (Fig. S5B). Age- and sex-matched littermates were used in the experiments. The housing, breeding and animal experiments were in accordance with the National Research Council’s Guide for the Care and Use of Laboratory Animals, using protocols approved by the Biological Research Ethics Committee of Shanghai Jiao Tong University.

LPS administration

Three-month-old *Saa3*-Tg mice and their respective WT littermates (n=4 per group, half males and half females) were given LPS at 15 mg/kg body weight intraperitoneally. Age-matched control mice were injected with equal volumes of saline. After 24 h, all mice were sacrificed by decapitation and their brains removed immediately for assays.

Immunofluorescence staining

The free-floating sections of mouse brain were processed for standard immunofluoresence staining. In brief, sections were incubated overnight at 4 ºC with anti-Saa3 (1:200) antibody and then incubated with Alex Fluor488-conjugated donkey anti-rabbit antibody (1:500, Invitrogen) at room temperature for 1 h. After another 3 washes in TBS, sections were stained for nuclei with 5 μg/ml of DAPI for 10 min at room temperature, and then mounted on glass slides. To identify the expression of Tau^pT205^ in neuron or astrocyte, sections were first stained with anti-MAP2 (1:200) or anti-GFAP-Cy3^TM^ (1:500) antibody overnight and then incubated in Alex Fluor568 donkey anti-mouse IgG (1:500; Invitrogen) for 1 h. The sections were rinsed in TBS and stained with anti-Tau^pT205^ antibody (1:200) overnight at 4ºC and incubated with Alex Fluor488 donkey anti-rabbit antibody (1:500; Invitrogen) before washing. The relative immunofluorescence intensity of Tau^pT205^ was quantified using the ImageProPlus Software (Media Cybernetics, Silver Spring, MD). The results were expressed as mean ± SEM, based on a minimum of 3 sections per animal and three 8-bit RGB digital images at the CA1 per animal (n=4 mice per group).

**LPS treatment *in vitro***

To detect Saa3 secretion into neuronal medium, primary neuronal cells derived from newborn *Saa3*-Tg pups and their WT littermates (non-Tg) were stimulated with L- LPS (0.1μg/m), H-LPS (1μg/ml) or PBS for 24 h. The medium was collected for ELISA.

**ELISA assay**

Saa3 secreted into the neuronal medium was measured by ELISA. Briefly, 100 μl neuronal medium was immobilized to ELISA plates at 4 ºC overnight, blocked with 3% BSA in PBS for 2 h at 37 ºC and then incubated with 20 μg/ml of the anti-Saa3 antibody at 4 ºC overnight. After washing 3 times with PBST (PBS + 0.5% Tween-20), 100 μl of a horseradish peroxidase-conjugated anti-rabbit IgG (1:500; Cell Signaling Technology, Beverly, MA) was incubated at 37 ºC for 90 min. The plate was emptied and washed 3 times with PBST, and 100 μl of the substrate mixture TMB/H_2_O_2_ was pipetted into each well after drying and incubation in 37 ºC for 10 min. The enzymatic reaction was terminated by adding 30 μl of 2 M H_2_SO_4_ per well. The concentration of Saa3 was determined by measurement of absorbance at 450 nm. The experiments were repeated for at least 3 times
