# Supplementary figures and images for: Suppression of LPS-induced tau hyperphosphorylation by serum amyloid A
Source: J Neuroinflammation. 2016 Feb 2;13:28. doi: 10.1186/s12974-016-0493-y (PMC4736117; doi:10.1186/s12974-016-0493-y)

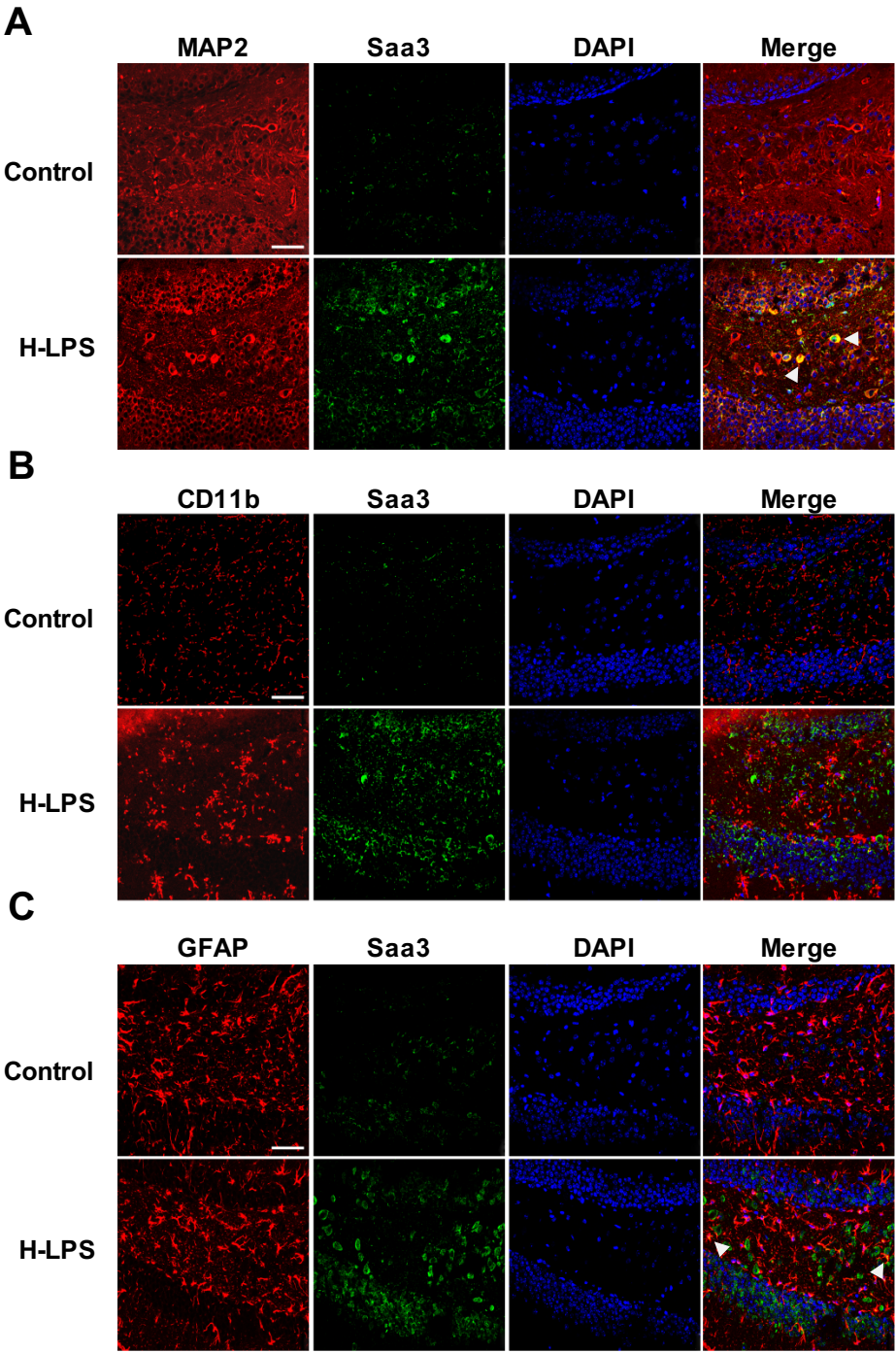

**A**

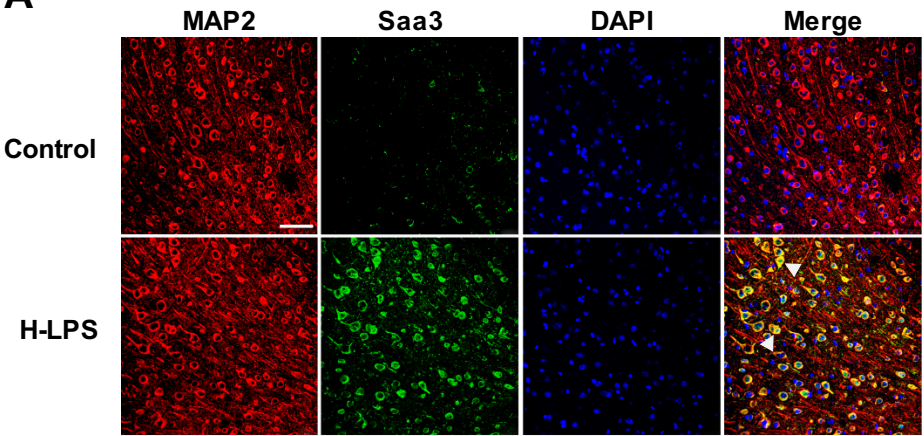

**B**

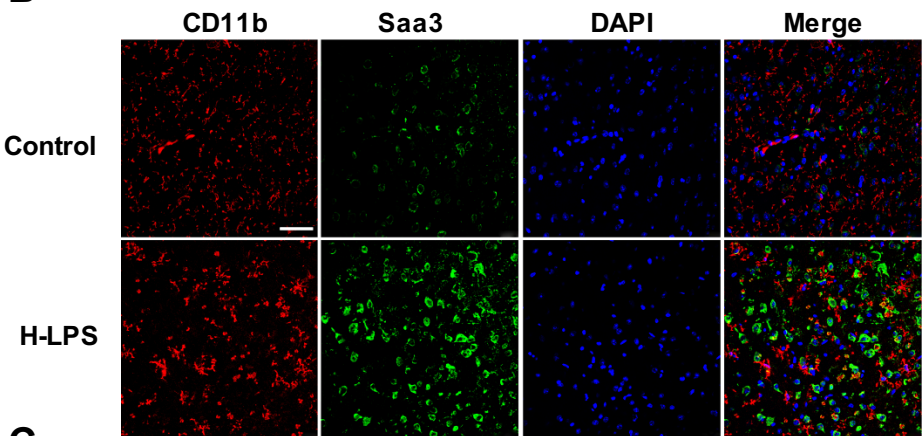

**C**

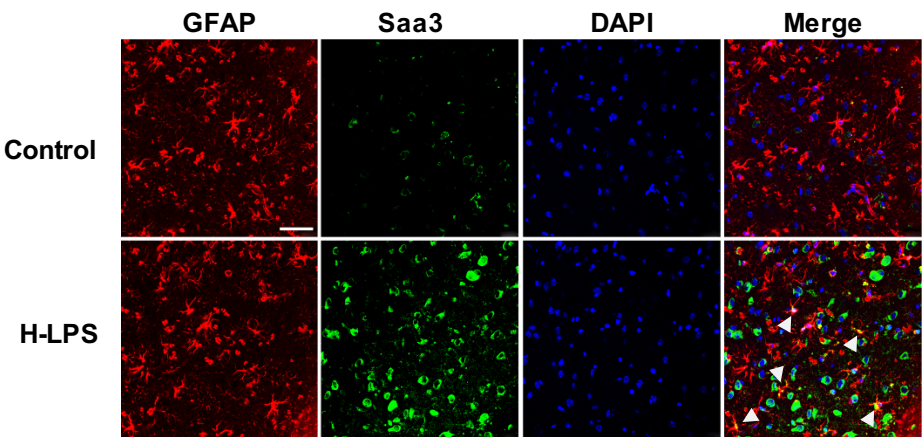

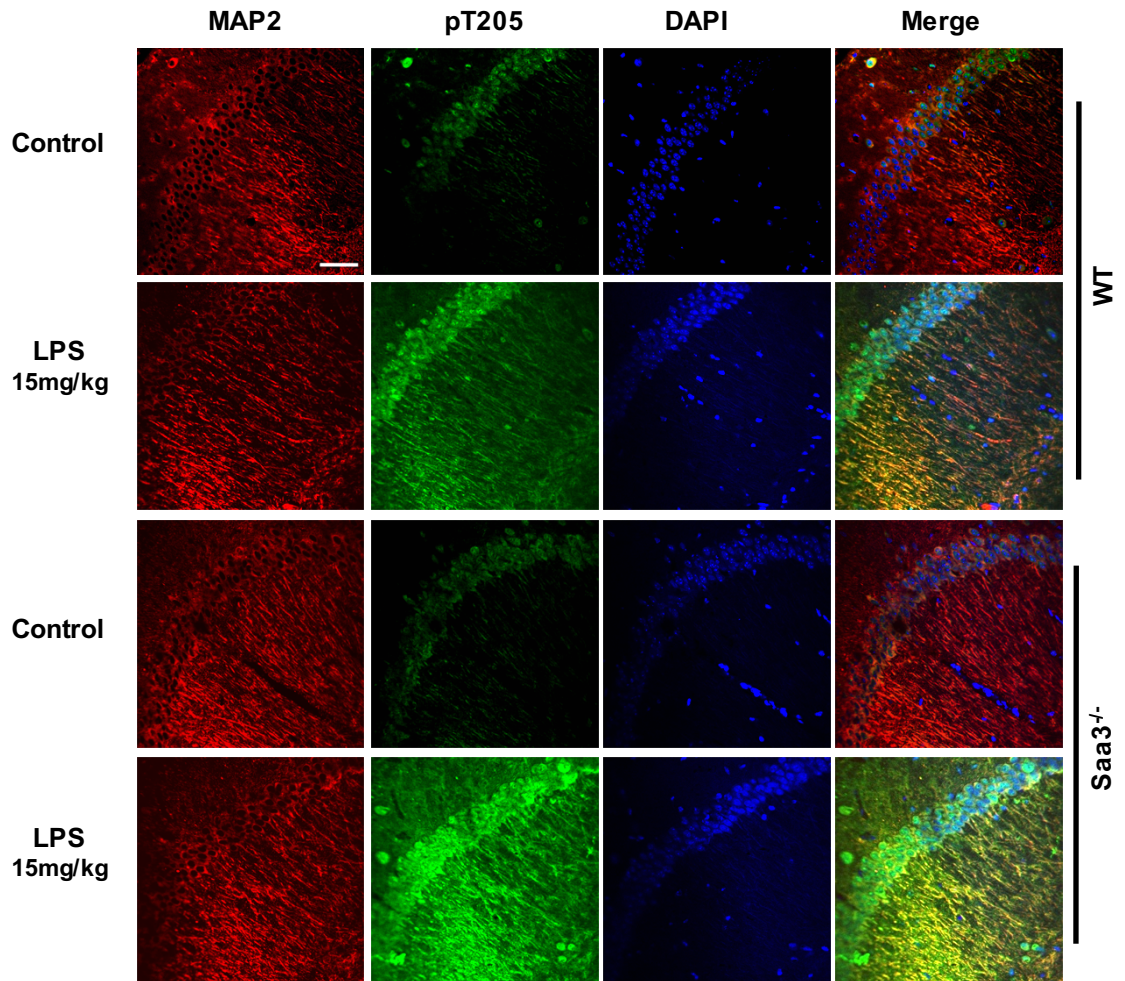

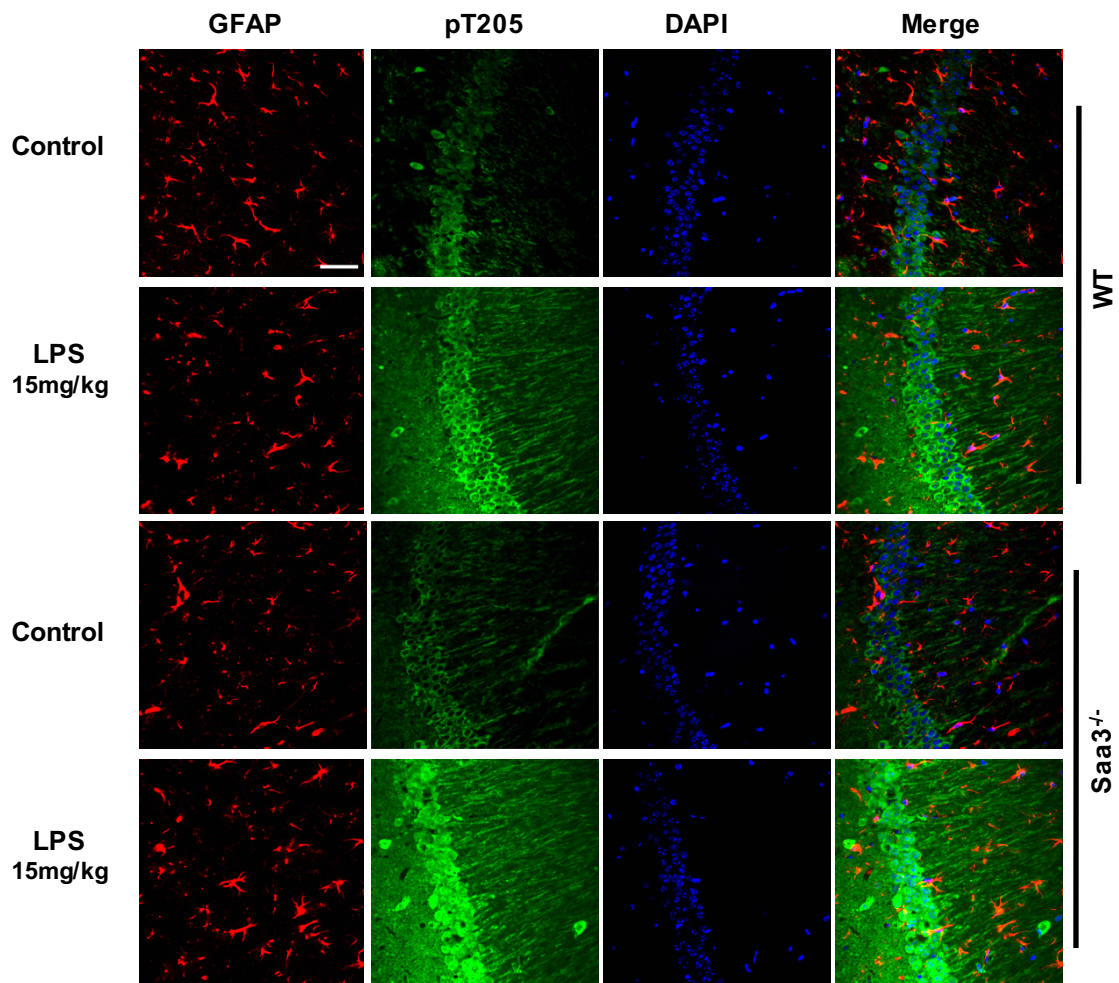

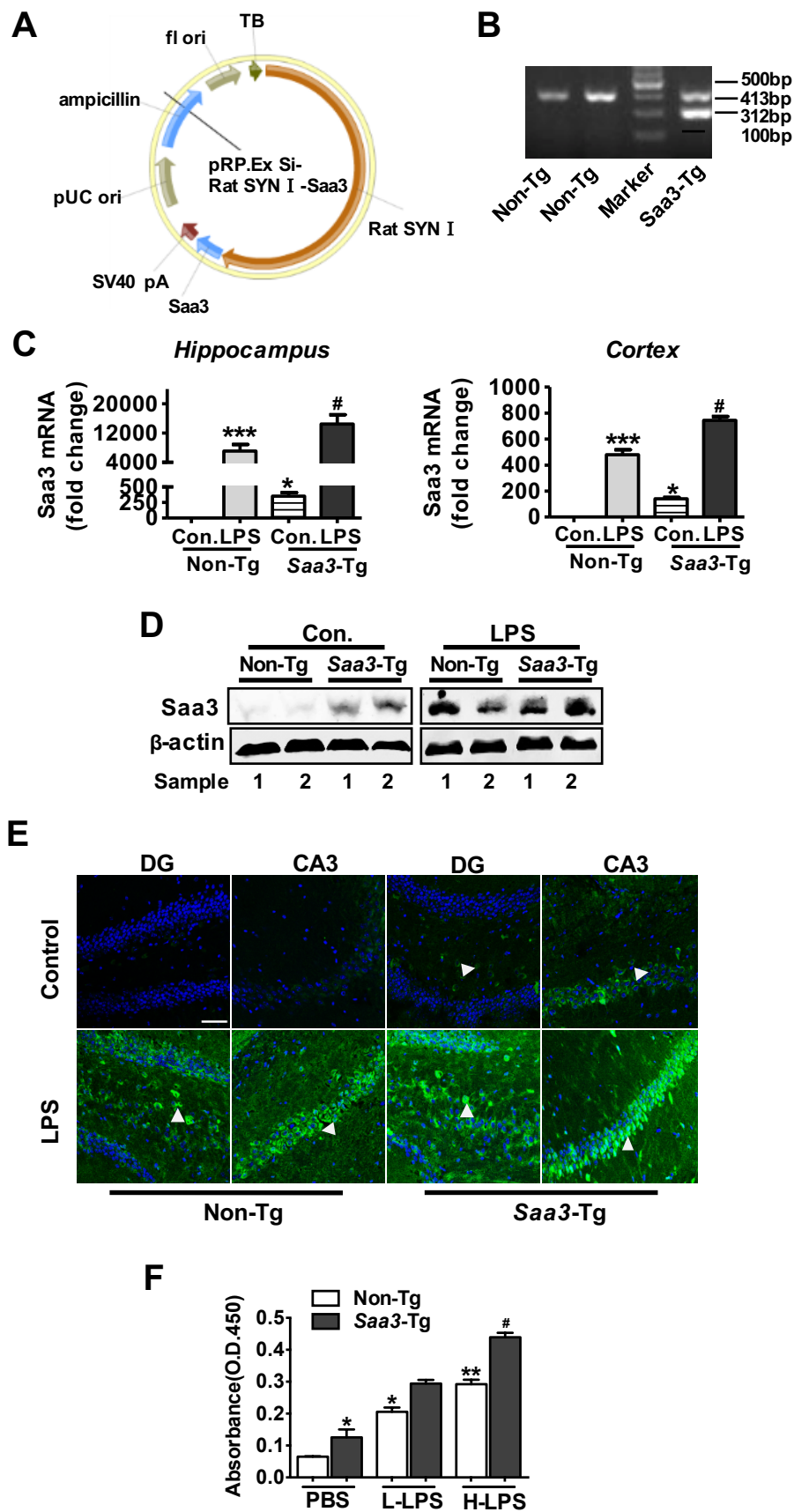

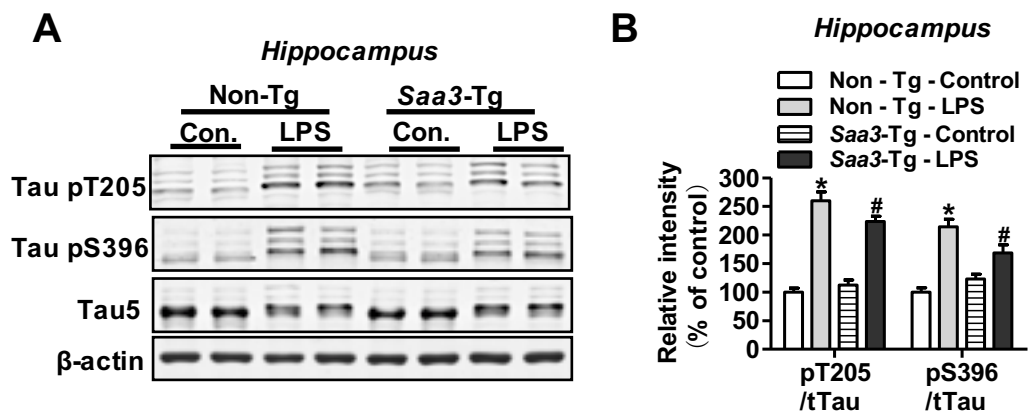

Supplement: Supplementary file 3 — This contains Figures S1–S6. This file contains 6 supplementary figures, listed as Figures S1 to S6, that provide the reader with additional information related to the main text of the paper. [file 12974_2016_493_MOESM3_ESM.pdf]
